# A New Method for Intense Cavitation Bubble Generation on Layer-by-Layer Assembled SLIPS

**DOI:** 10.1038/s41598-019-48175-4

**Published:** 2019-08-12

**Authors:** Araz Sheibani Aghdam, Morteza Ghorbani, Gokberk Deprem, Fevzi Çakmak Cebeci, Ali Koşar

**Affiliations:** 1 Faculty of Engineering and Natural Sciences, Sabanci University, Tuzla, Istanbul 34956 Turkey; 2 Mechatronics Engineering Program, Faculty of Engineering and Natural Science, Sabanci University, Istanbul, 34956 Turkey; 3 Department of Biomedical Engineering and Health Systems, KTH Royal Institute of Technology, Stockholm, Sweden; 4 Center of Excellence for Functional Surfaces and Interfaces for Nano-Diagnostics (EFSUN), Sabanci University, Istanbul, 34956 Turkey; 5 Sabanci University SUNUM Nanotechnology Research and Application Center, Istanbul, 34956 Turkey

**Keywords:** Mechanical engineering, Nanoscale materials

## Abstract

The importance of surface topology for the generation of cavitating flows in micro scale has been emphasized during the last decade. In this regard, the utilization of surface roughness elements is not only beneficial in promoting mass transportation mechanisms, but also in improving the surface characteristics by offering new interacting surface areas. Therefore, it is possible to increase the performance of microfluidic systems involving multiphase flows via modifying the surface. In this study, we aim to enhance generation and intensification of cavitating flows inside microfluidic devices by developing artificial roughness elements and trapping hydrophobic fluorinated lubricants. For this, we employed different microfluidic devices with various hydraulic diameters, while roughness structures with different lengths were formed on the side walls of microchannel configurations. The surface roughness of these devices was developed by assembling various sizes of silica nanoparticles using the layer-by-layer technique (D2). In addition, to compare the cavitating flow intensity with regular devices having plain surfaces (D1), highly fluorinated oil was trapped within the pores of the existing thin films in the configuration D2 via providing the Slippery Liquid-Infused Porous Surface (D3). The microfluidic devices housing the short microchannel and the extended channel were exposed to upstream pressures varying from 1 to 7.23 MPa. Cavitation inception and supercavitation condition occured at much lower upstream pressures for the configurations of D2 and D3. Interestingly, hydraulic flip, which rarely appears in the conventional conical nozzles at high pressures, was observed at moderate upstream pressures for the configuration D2 proving the air passage existence along one side of the channel wall.

## Introduction

The nature has inspired the humanity in the development of most of the cutting edge technologies to cope with different problems to improve the life quality. During recent years, lotus leaf and nepenthes pitcher-plant, which offer outstanding natural designs involving liquids and gases, have played a crucial role in inspiring the scientists to develop Slippery Liquid-Infused Porous Surfaces (SLIPS). These types of enhanced surfaces are able to control and improve different surface properties such as hydrophobicity and icephobicity for the use in separation, protection and transportation of underwater macro gas bubbles^1,2^.

Hydrodynamic cavitation in microscale as an emerging topic in the field of small bubble generation has attracted considerable attention in the engineering, energy and biomedical communities. This phenomenon can be manipulated by surface modifications in such a way that more intense cavity clouds could be generated. Recently, it was shown that surface roughness apart from the working fluid inside microfluidic devices has a significant effect on the generation of cavitation bubbles^3^. Therefore, it is of great importance to investigate the surface characteristics by focusing on substrate nanoengineering techniques to achieve intense bubble generation and earlier cavitation inception, which could be utilized in energy and biomedical applications.

SLIPS can cover rough surfaces prepared on a substrate, which are formed using various techniques such as carving using chemical or physical reactions, attachment of nanoparticles (NPs), rods or wires using deposition, casting or assembly techniques. The resultant pores within the surface of the substrate are used as reservoirs to trap a slippery lubricant. Thus, changing the characteristics of rough surfaces can provide proper locations to trap different types of hydro or oleo phobic lubricants within pores and valleys of the surface. During the last decade, layer-by-layer (LbL) assembly as an emerging technique has attracted the interest of many scientists for achieving different substrates regarding the surface roughness^4–6^. This method has been introduced as a sub-nano scale approach in the assembly of any kind of electrostatically charged particles of electrolytes on oppositely charged substrates or sublayers^7^. The self-assembly of alternately charged polyelectrolytes as adhesion layer can provide a reliable and adhesive sublayer, which is sufficiently charged to embrace oppositely charged NPs and to attain a robust and mechanically strong and rough surface. As a result, the lubricant trapped within these porosities serves as stable and endurable SLIPS^8^.

The studies related to the surface roughness effect on bubble dynamics mostly focused on bubble separation and mass transportation. In this regard, SLIPS, hydrophobic and superhydrophobic surfaces have been considered by scientists in trapping, separating and transporting air bubbles under water. There is a major difference in the behavior of these surfaces encountering bubbles. For hydrophobic and surperhydrophobic surfaces, the micro and nanoscale roughness on the substrate traps pockets of air within its pores and does not allow water to penetrate. On the other hand, SLIPS provide hydrophobicity and aerophilicity without trapping dry air pocket, where water droplet in air environment or air bubble under water becomes confined under a thin layer of the trapped lubricant and not only withstands the upward buoyancy force of the air bubble but also increases the life time and durability of air bubbles to pressure changes^2,9^. Some researchers have taken the advantages of SLIPS to transport bubbles using the buoyancy force, to change the path of bubble and deviate their route, to absorb bubbles generated within a liquid and direct them along a trajectory^10–12^. The functionalities of SLIPS in protecting gas bubbles and increasing their life time under water have attracted the attention of related research communities dealing with macro bubbles. However, the behavior of these surfaces interacting with micro size bubbles clusters and clouds has not been sufficiently covered.

Few attempts regarding hydrodynamic cavitation have been made to study cavitating flows in micro scale in the literature^13,14^, where advanced surface engineering techniques were not considered. It could be possible to reach fully developed supercavitation conditions inside microfluidic devices^15,16^ and to employ the energy released from the collapse of cavitation bubbles in different applications varying from thermoelectric energy generation^17^ to sonoluminescence applications^18^. Therefore, in the light of the studies on cavitation in microscale, the recent investigations deal with the surface configurations such as roughness implementations and embedded pillars inside a microchannel^19^ to generate intensified cavitating flows.

Motivated by the promising results of recent studies, SLIPS could be achieved on the microchannels in this study by assembling several layers of thin film using the LbL technique, and fluorinated oil was trapped within the pores of the film. The microfluidic devices were initially fabricated using a state-of-the-art process flow for formation of side wall roughened channels. Several layers of polyelectrolytes and NPs were assembled on the surface of microchannels by using the LbL technique and then by making the thin film surface favorable for the lubricant. The porosity of the thin film was filled by the oil, and the performances of the enhanced microchannels were evaluated. The surface enhanced devices with different hydraulic diameters were exposed to upstream pressures ranging from 1 to 7.23 MPa, while the downstream pressure was kept at the atmospheric pressure level. Accordingly, to generate intense cavitating flows and to lead to earlier cavitation inception, the surface modification effects were examined. This study reveals the potential of combined roughened surfaces and SLIPS in generating cavitating flows (even at the center of the microchannel in some cases), which bolsters the idea of facile cavitating flow patterns in micro scale even at low pressure drops. The results of this study could be well exploited in energy harvesting applications in microscale as well as in biomedical applications.

## Results and Discussion

### Surface properties

**Figure 1:**
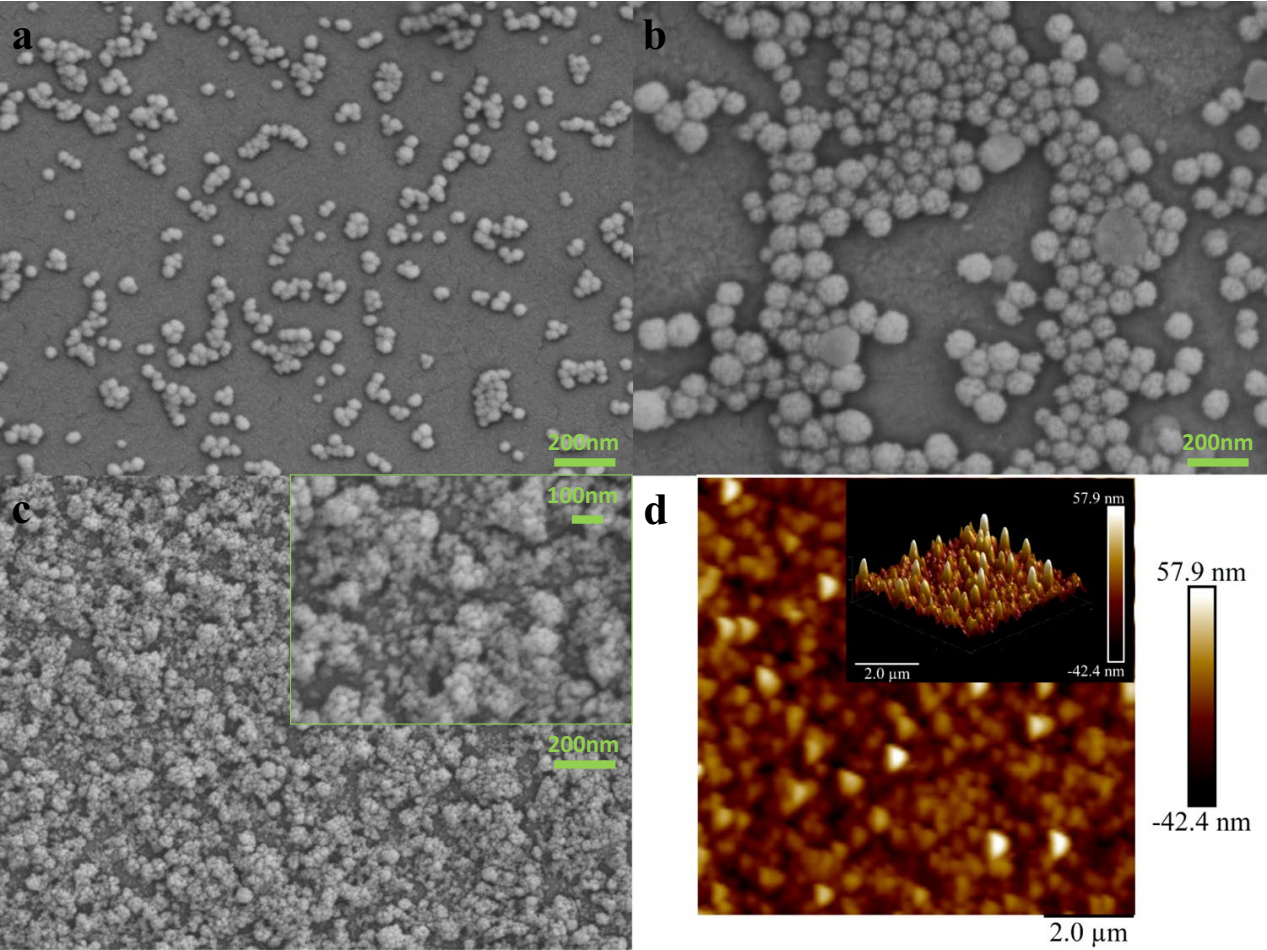
SEM and AFM images of synthesized silica nanoparticles and layer by layer assembled D2 channel a-40nm NPs b-80nm NPs c-layer by layer assembled D2 coating d- AFM height image of D2 coating.

Figure 1 displays the SEM images of silica nanoparticles and the D2 thin film on silicon wafer. The SEM images of NPs are consistent with the DLS results. The sizes of the nanoparticles were measured as 40 and 80 nm (Table 1). The mesoporous surface of the bigger nanoparticles is shown in Fig. 1b, which proves the nanoscale roughness and higher efficiency of the surface in making the infused lubricant become stable on the surface of samples. It is clear from Fig. 1c that a uniform layer of the combination of the two different sizes of the nanoparticles is assembled on the surface of the silicon wafer and creates artificial roughness on the smooth surface of the wafer.

**Table 1:** DLS size and Zeta potential of synthesized and mixed silica nanoparticles.

Sample number	DLS Size [nm]	Zeta potential [mV]	SEM Particle size [nm]	STD	[TEOS]/[NH3]aq
N1	38.2	−22.6	33.4	7.55	6.5
N2	86.5	−38.5	81.4	8.25	3.5
N1, N2 (1:1)	68.1	−35.0	—		—

The Bruggeman effective medium approximation (EMA) was used for modelling ellipsometry results of porous surfaces to estimate the thickness and fraction of the voids within the film^20^. The thickness of the layer by layer assembled thin film was measured as 120 nm, and the 10% EMA of the voids within the film can be considered as another evidence for the existence of the pores to save the lubricant and for the formation of the SLIPS.

Introducing PFDTS reduces the surface energy, which leads to hydrophilicity. The Fluorinated compound alters the affinity of the surface, and the surface roughness provided by the thin film makes the pores hold the lubricant on the surface under harsh conditions. Therefore, the lubricant withstands intensified cavitating flows even at very high velocities and turbulent flow conditions, and the flow patterns do not change during the multiple and prolonged experiments suggesting the repeatability of the results. The Root Mean Square (RMS) of the roughness of the surface is reported as 14.2 nm according to the AFM results. The projected surface of the thin film shows a 0.6% increase in the surface area, which ensures that the lubricant has some space within the pores of the surface to build a reservoir and be caught there during the fluid flow.

### The effect of the SLIPS and LBL assembled silica nanoparticles on the inception of the cavitation phenomenon

**Figure 2:**
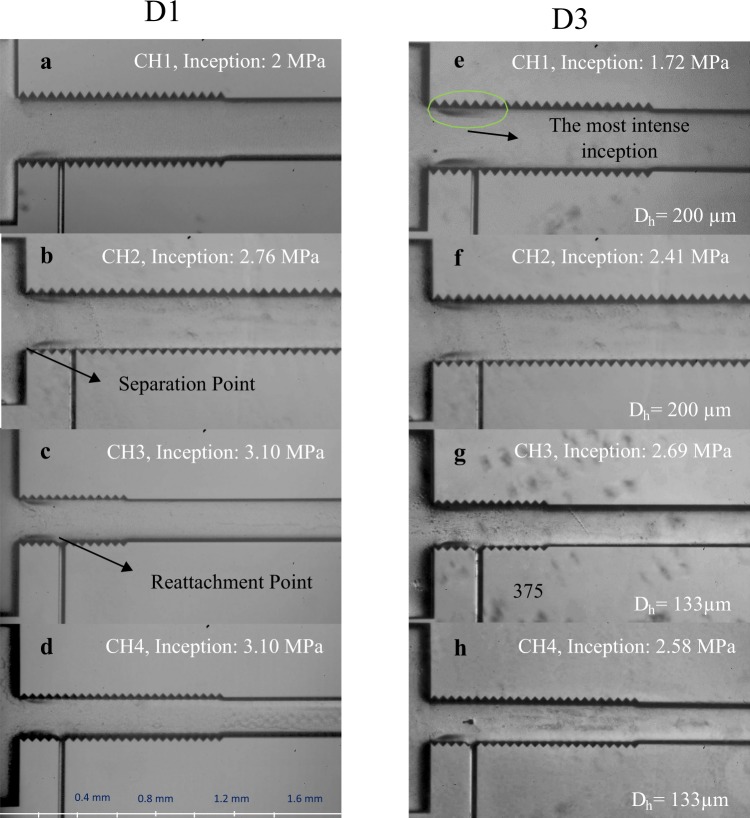
The qualitative comparison of the inception of cavitation phenomenon inside the regular microchannels with state-of-the–art design (D1) and SLIPS enhanced one (D3).

Figure 2 displays a general overview of cavitation inception. Just beyond the vena contracta, the pressure suddenly decreases, and cavitation bubbles appear at each column. The inception of cavitating flows was investigated thoroughly inside the microfluidic devices using the fluorinated enhanced surface by LBL assembled silica nanoparticles and SLIPS, which were named in this study as D2 and D3, respectively. The inception was visualized in two regions, namely microchannel and the extended channel, which is the channel downstream of the microchannel. The results obtained from these devices were compared with the regular device with plain surface, which is called D1 in this study.

Figure 2 illustrates the effect of the SLIPS (D3) in the generation of cavitation bubbles at lower pressures. The results obtained from the devices with D3 coating are compared with the results of the devices having plain surfaces in this figure. The inception pressure is reduced more in the smaller channels (e.g. CH4), while there is still a considerable decrease in the inception pressure for the bigger channels (e.g. CH1) with intensified cavitating flows. Surface roughness is one of the major parameters influencing cavitating flows. It affects the boundary layer, which in turn affects the occurrence of cavitating flows. The surface roughness facilitates the cavitation inception and augments the nucleation of cavitation bubbles, since heterogeneous nucleation occurs in small scale phase change phenomena. Thus, crevices, roughness elements and porosity facilitate nucleation and facilitates cavitation inception. On the other hand, it can increase the flow instability, depending on the size and spatial distribution of the roughness elements^21^.

The overall trend in the cavitation inception in the regular side wall roughened channels is reflected as lower upstream pressure values for the bigger channels, which shows an earlier arrival of the vapor saturation pressure condition in these channels (e.g. CH1). Therefore, besides the new structural design, SLIPS is considered as an effective way to lead to an earlier generation of cavitation, which is essential to decrease the input energy and to increase the efficiency of microfluidic devices in providing cavitating flows.

Cavitation inception occurs with the formation of attached twin cavities inside the microchannel, which are limited to the inlet of the nozzle. The most important parameter in the formation of the flow regimes in micro scale is the surface tension. The surface tension of water is s = 0.0728 N/m at 293.15 K, and the previous results corresponding to water indicated that bubbly flow is a less dominant flow regime for micro scale cavitating flows^13^. Weak forces due to surface tension make the bubbles in liquids grow more, and the surface nuclei are the source for them. In addition, the surface tension mostly determines the bubble departure and prevents the detachment of the voids/non condensable gases from the surface. Since the diameter of the bubble is more comparable to the diameter of the channel in micro scale, this effect is more pronounced in the microfluidic devices facilitating the condition for the generation of cavitating flows. Therefore, cavitation phenomenon appears in bubbly flow pattern inside the microchannel within a small range of working conditions, and twin cavity clouds appear instead.

**Figure 3:**
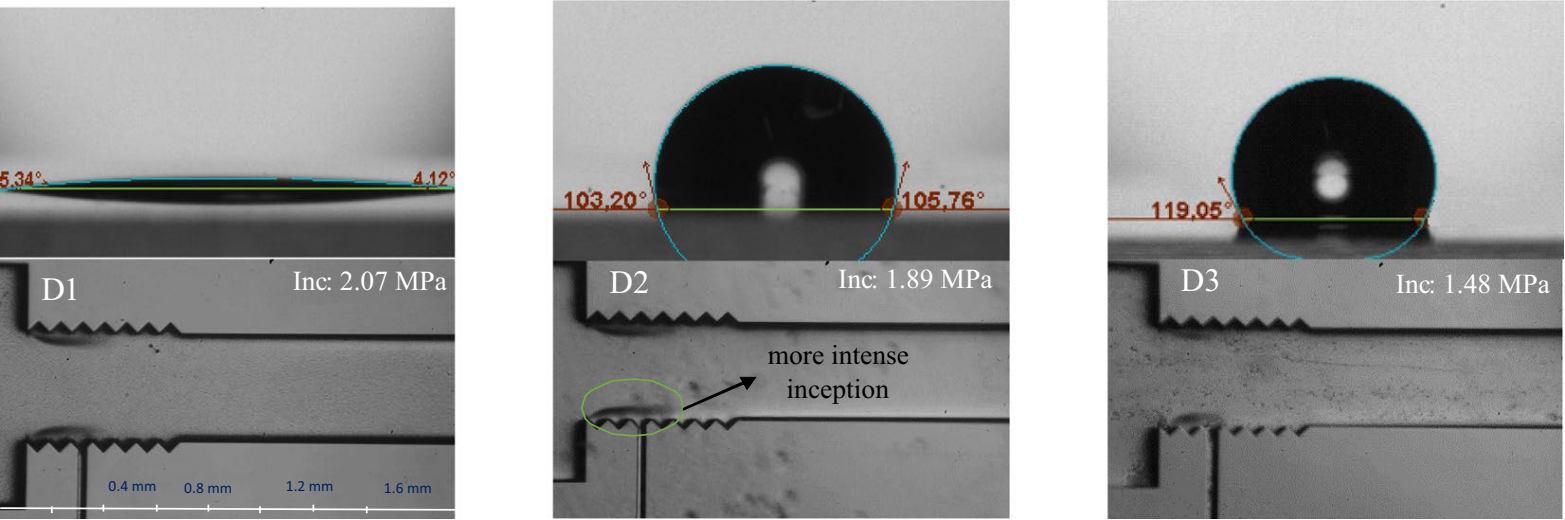
The inception inside CH7 and the enhancement demonstration with SLIPS and LBL silica nanoparticles and the water contact angle of the channel a- hydrophilic surface of D1 coating b- hydrophobic surface of D2 coating c- hydrophobic surface of the D3 coating and thin layer of lubricant covering the droplet.

To reveal the effect of surface modification on twin cavity formations, more tests were done on the biggest available microchannel (CH7), where the side wall roughness elements cover one third of the microchannel. Figure 3 shows the inception in CH7 with the coatings of D2 and D3 and their comparison with the plain microchannel. As seen, the lubricant is deflected due to the high pressure fluid flow, and the roughness of the surface has a direct contact with the flow. As a result, the inception pressure decreases to 1.48 MPa for the coating D3 corresponding to the lowest value for inception pressure. For the D3 coating, after nucleation of cavitation bubbles, a very thin layer of lubricant covers the surface of the bubble and protects it from collapse^22^. However, D2 samples do not have any lubricant within its pores. Thus, it is shown that larger microchannels with shorter side wall roughness elements cause cavitation inception at much lower upstream pressure, which is a result of surface dominating and dictating cavitation events.

Contact angle measurements show that water has the highest contact angle in the case of D3 coating. The contact angle hysteresis of the D2 and D3 coatings are measured to be 100 ° and 5 °, respectively, which means that the droplet on the lubricant of the D3 coating can easily slide. However, the droplet on the D2 coating is pinned on the surface^23^. On the other hand, the D1 surface is hydrophilic, and the water droplet is completely spread on the surface. It should be noted that the behavior of contact angle of air bubble in water medium can be predicted using the water droplet in air medium. They can be considered as supplementary angles as documented in the literature^11^. The curved shape of the intersection of the droplet and the lubricant for the D3 coating shows that a very thin layer of the lubricant covers the surface of the droplet, which can protect the bubble from collapse in water medium^9,22^.

**Figure 4:**
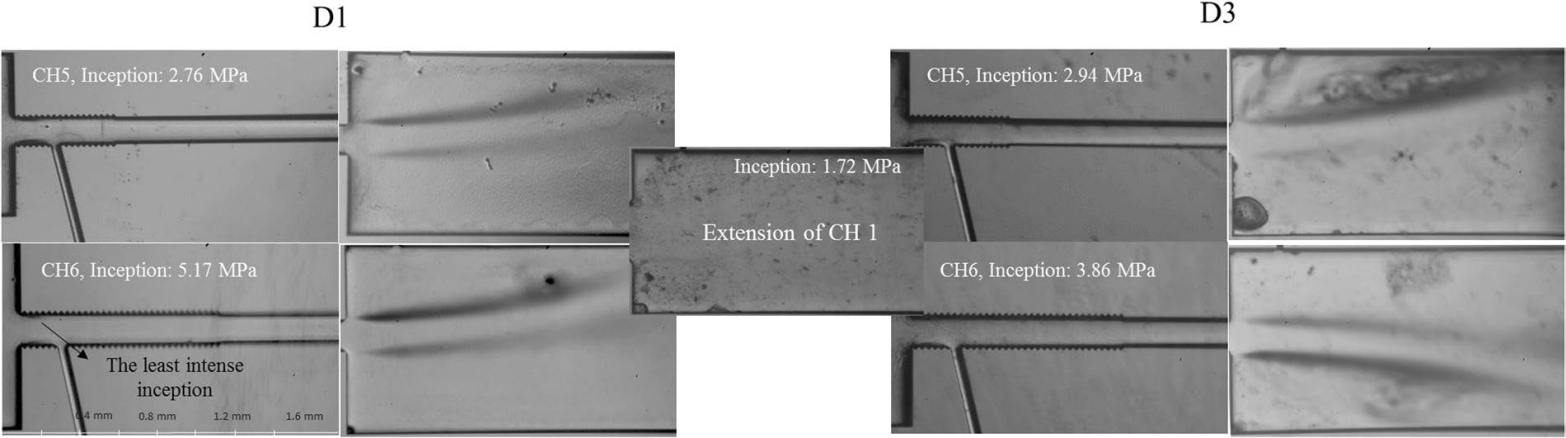
Cavitation inception in the extended channel for the smaller microchannels (CH5 and 6) enhanced by D3 and the comparison with the corresponding extended channel for the larger microchannel.

The reduction in the hydraulic diameter of the microchannel leads to an interesting behavior in the inception and flow patterns inside the microchannel and the extended channel. While weaker inception is observed inside the smaller microchannel (CH5 and 6), much more intense inception is captured in the extended channel. Interestingly, there are almost no twin cavity clouds in the extended channel for the bigger microchannels at corresponding inception pressures as shown in Fig. 4.

**Figure 5:**
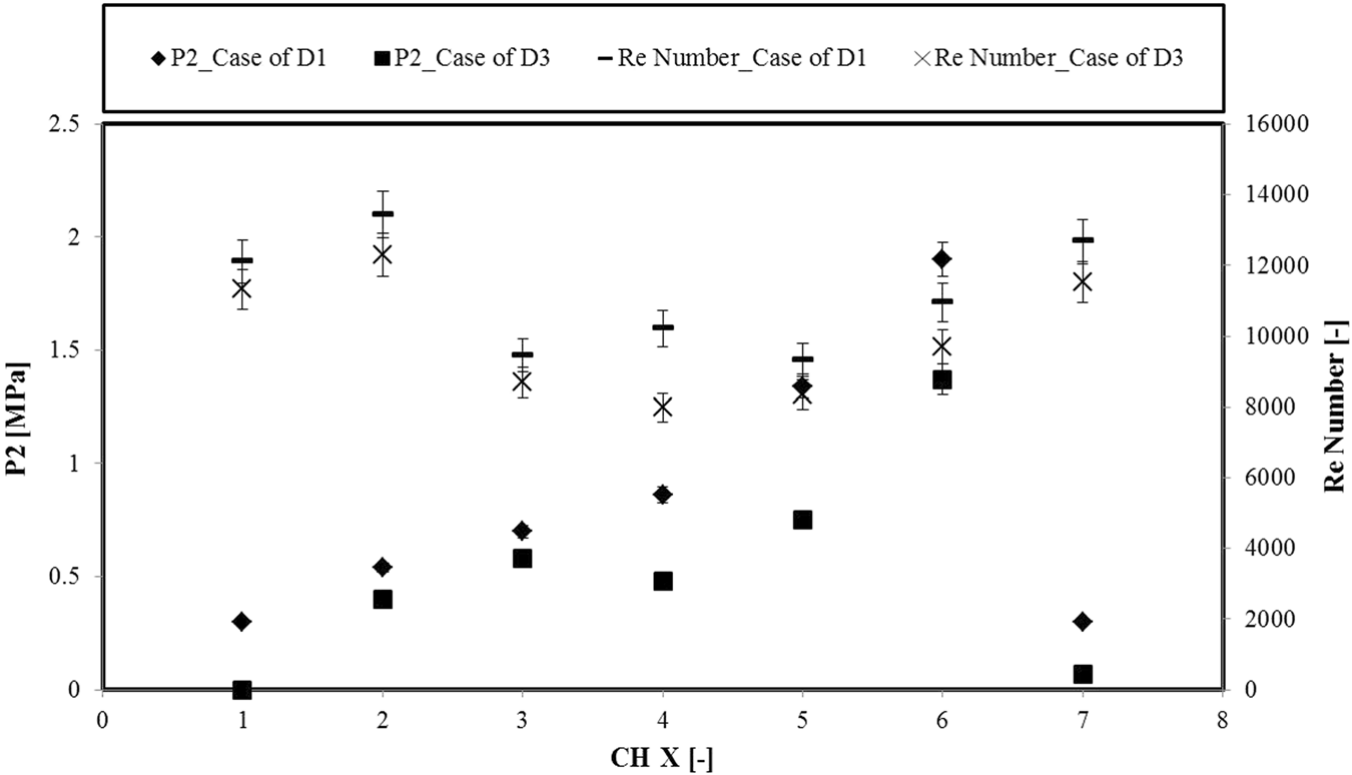
The variations in pressure at the location of vena contracta (P2) and Reynolds number for the cases of D1 and D3 at cavitation inception for different microchannels (X stands for the number of the channels).

Cavitation occurs due to rapid changes at pressure in the smaller microchannels, when flow is injected inside extended channel. This rapid change of channel size has a direct effect on both pressure and velocity, which causes cavitation, and bubbles are mostly visible in the area such as vena contracta. The high values of the pressure at the vena contracta location (P2) for smaller microchannels support the hypothesis of later occurrence of inception (Fig. 5). In addition, the pressure in the extended channel exhibits a stable trend (P_abs_ = 0), which is less compared to the larger microchannels. This proves that the design parameters are significant in achieving the desirable cavitation feature at different locations. Apart from the size variation effects, the surface enhancement with the coating D3 also exhibits its significant effect in the generation of more intense clouds in the extended channel. Large vortices and several separations are observed as shown in Fig. 4 for the D3 coating, which presents larger pressure variations for this case. The movement and direction of flow depends on several parameters. When there are bends in either upstream or downstream within the microchannel or extended channel, they make the flow move upward or downward depending on the position and angle of the bend before the channel. There are also some other phenomena such as step flow, and the wake region at the corner of the channel can lead to periodic movements of the jet with low frequency due to separation effects (similar to Karman vortex phenomenon). The detailed comparison of the cavitation inception between plain microchannel and D3 coating for smaller channels is shown in the Fig. 4.

Figure 5 shows the pressures at the location of the vena contracta (P2) and Reynolds number (${\rm{Re}}={{\rm{\rho }}v}_{{\rm{ref}}}{{\rm{D}}}_{{\rm{h}}}/{\rm{\mu }}$, where v_ref_ is the flow velocity and D_h_ is the hydraulic diameter), at cavitation inception for the surface configurations D1 and D3. The results illustrate that the P2 has a lower value for all the microchannels (CH X-here X stands for the number of the channels) for the coating D3 in comparison to the D1 device. The reduction in P2 becomes more for smaller microchannels for the coating D3 indicating the effect of the SLIPS on the inception and generation of the bubbles in larger channels. As seen, the results with SLIPS are more favorable in terms of earlier inception in comparison to the smaller channels.

The results of the Reynolds number also present a decreasing trend with the introduction of SLIPS coating to the microchannels, which implies earlier cavitation inception for the D3 coating. Furthermore, the Reynolds number values suggest turbulent flow conditions for all the cases, while its values for the smaller channels have smaller magnitudes. This suggests that the velocity of cavitating flows at the inception has higher values for the larger microchannels despite the lower pressure magnitudes compared to the smaller microchannels. As a result, the larger microchannel with D3 coating results in the most intense cavitation inception.

### The effect of the LbL assembled SLIPS on the development of the cavitating flow

**Figure 6:**
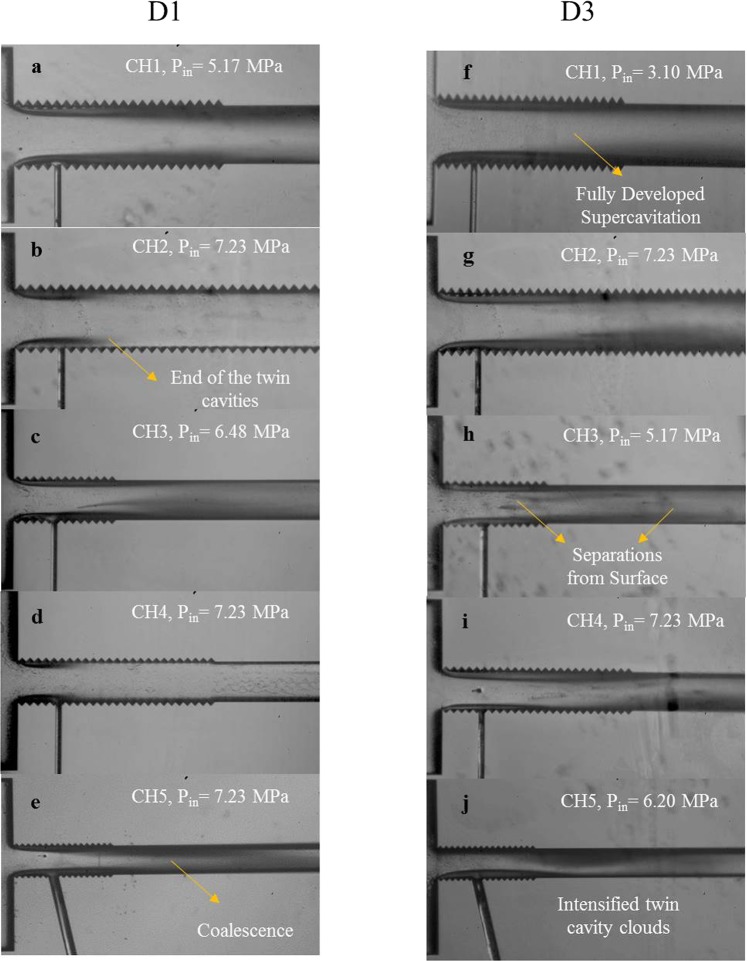
The comparison in the supercavitation flow regime inside different microchannels for the D3 coating with plain D1 microchannels.

Figure 6 presents a qualitative comparison of flow pattern variations inside different microchannels for the D1 and D3 cases. The results show that the supercavitation condition exists for all the microchannels for the D3 coating, while this flow pattern is not visible in the case of D1 for some of the microchannels including the larger ones (e.g. CH2 and CH4). In addition, the supercavitation flow pattern appears at much lower upstream pressure values for the case of D3.

According to Fig. 6, the large reduction in pressures to achieve supercavitation flows can be observed in the larger microchannels (CH1). Moreover, the results illustrate that for the longer side wall roughness elements with a reduction in the hydraulic diameter (CH4) for the D1 device, supercavitation cannot be seen even at high upstream pressure (7.23 MPa). However, supercavitation flow regime with intensified cavitating flows (even from the center of the channel) is observed in the smaller microchannels for the D3 device. This flow regime is also visualized for other channels, while the cavitation cloud generation from the center of the channel is observed in CH5 with a more intense behavior for the D3 device. This outcome demonstrates the effectiveness of the roughness elements in generating intense cavitation features in the micro scale. On the contrary, the roughness elements in the conventional channels are not as effective as in the microchannels^24^. As a result, along with the pressure rise, the roughness distribution induces intense turbulent flow conditions, which are accompanied with high surface tension in micro scale, providing an interesting flow regime filling almost the entire microchannel.

**Figure 7:**
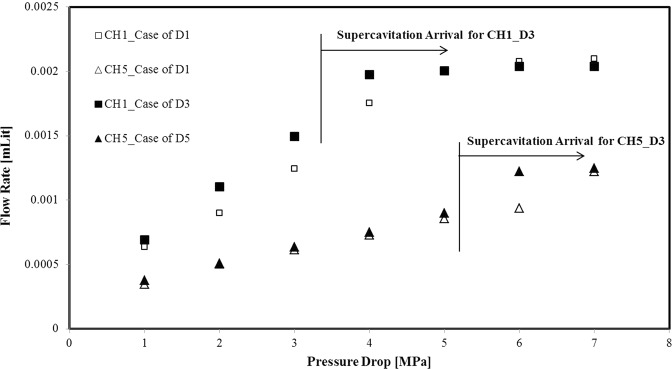
The flow rate variations in CH1 and CH5 as a function of pressure drop.

Figure 7 presents the flow rate variations for CH1 and CH5 with respect to the pressure drop. Flow rate has a constant value beyond the pressure drop of 3.9 MPa for the D3 coating, which marks the arrival of the supercavitation condition, while the flow rate is constant beyond the pressure drop of 5.9 MPa implying later arrival of the supercavitation condition in CH5.

**Figure 8:**
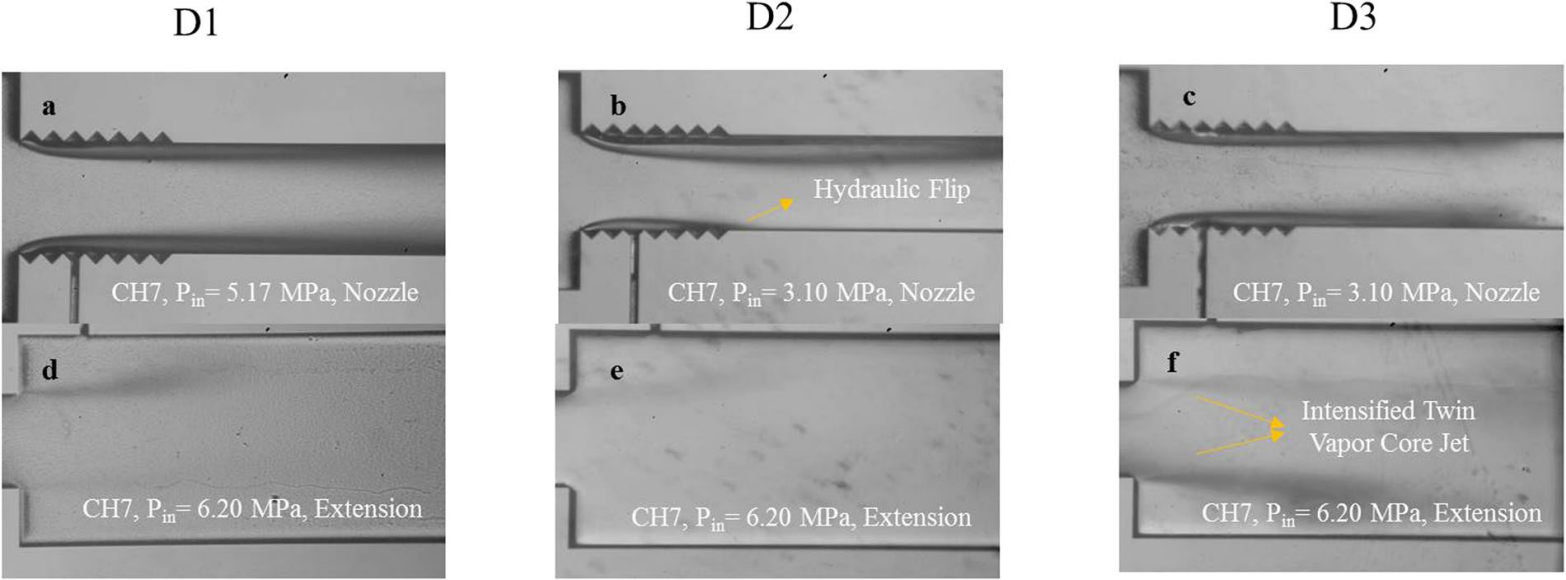
The cavitating flow patterns inside the microchannel and extended channel of the CH7 device for the configuration D1 and D2 and D3 coatings.

The cavitating flow patterns in CH7 for the D1 device, and D2 and D3 coatings are shown in Fig. 8, which displays the effect of the modified surface and the trapped lubricant on the bubble nucleation and following growth. Generated bubbles follow different paths to reach the extended channel and the targeted wall. Most of the cavitation bubbles survive and reach the extension part by passing through the liquid without touching the walls or the nozzle surface. The roughness elements provide favorable locations at the throat and even within the nozzle for nucleation of bubbles and also increase the probability of collapse in the cases of contact of the bubble with the surface or the walls of the nozzle. Lower inception and lower supercavitation pressures in D2 devices in comparison with the D1 devices prove that generation of bubbles in D2 devices is significantly enhanced and despite the collapse of the bubbles in the nozzle, supercavitation can be observed at the pressure 3.10 MPa (Fig. 8). Comparing the results in the extended regions of D1 and D2 devices at 6.20 MPa reveals that the number of the survived bubbles is almost the same. This means that most of the bubbles generated inside D2 channels collapse. As the only difference between D1 and D2 devices is the roughness of the surface, the roughness of the surface in the channel not only increases the amount of generated bubbles but also plays a dominant role in the collapse of the bubbles. Although D1 devices generate lower amount of bubbles, most of them survive either by passing directly through the fluid or by bouncing the surface without rupture. The chance of the survival of the cavitation bubbles can be clearly visualized from the extension region. For instance, the extension region of CH7 channels at upstream pressure 5.17 MPa is presented in Fig. 8. It is obvious that the bubbles in D2 devices collapse before reaching the extension region and D3 devices protect the bubbles from collapse and enhance the delivery and the density of the bubble cloud to the extension region.

Introducing the lubricant to the devices and trapping it within the pores of D2 devices and developing SLIPS D3 devices make the surface smoother and more favorable for the cavitation bubble protection. Tang *et al*.^9^ claims that the lubricant wraps the surface of the bubble with a very thin layer of oil and protects it from pressure fluctuations or rough conditions. The colliding bubbles with the surface of SLIPS are pinned onto the surface, and the cloak-wise layer of the lubricant lowers the chance of collapse^9,12^. Therefore, the cavitation bubbles survive along the channel toward the extension region, and bubble delivery can be achieved.

Figure 8 shows a thorough comparison of the flow transition inside CH7 for the cases of D1, D2 and D3. This figure presents the effect of the SLIPS coating in reaching the fully developed supercavitation condition at a low upstream pressure. While the supercavitation is observed at the upstream pressure of 3.10 MPa for the D3 coating, separated cavity clouds are also observed at the higher upstream pressure (6.20 MPa) from the center of the microchannel although CH7 is relatively large in terms of hydraulic diameter. Another interesting pattern is observed for the upstream pressure higher than 4.13 MPa for the D2 coating, which is called hydraulic flip. It is a rare pattern, which happens when the cavity cloud reaches the outlet of the microchannel, and the air flow existing at the extended channel tries to find a way in order to fill the entire boundary of the channel. This backflow occurs for the D2 coating implying the existence of the air passage replacing the main jet flow from the wall of the microchannel. During this phenomenon, the water jet is separated from the cavitating flow area and is of great importance in different industrial applications such as hydro-entangling, where uniform fibers form via water jets. To our best knowledge, hydraulic flip is seen for the first time in micro scale in this study.

**Figure 9:**
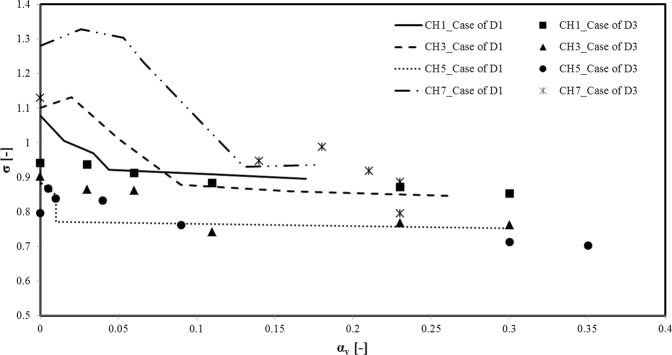
The variations in cavitation number as a function of vapor volume fraction in different microchannels for the D1 and D3 cases.

**Figure 10:**
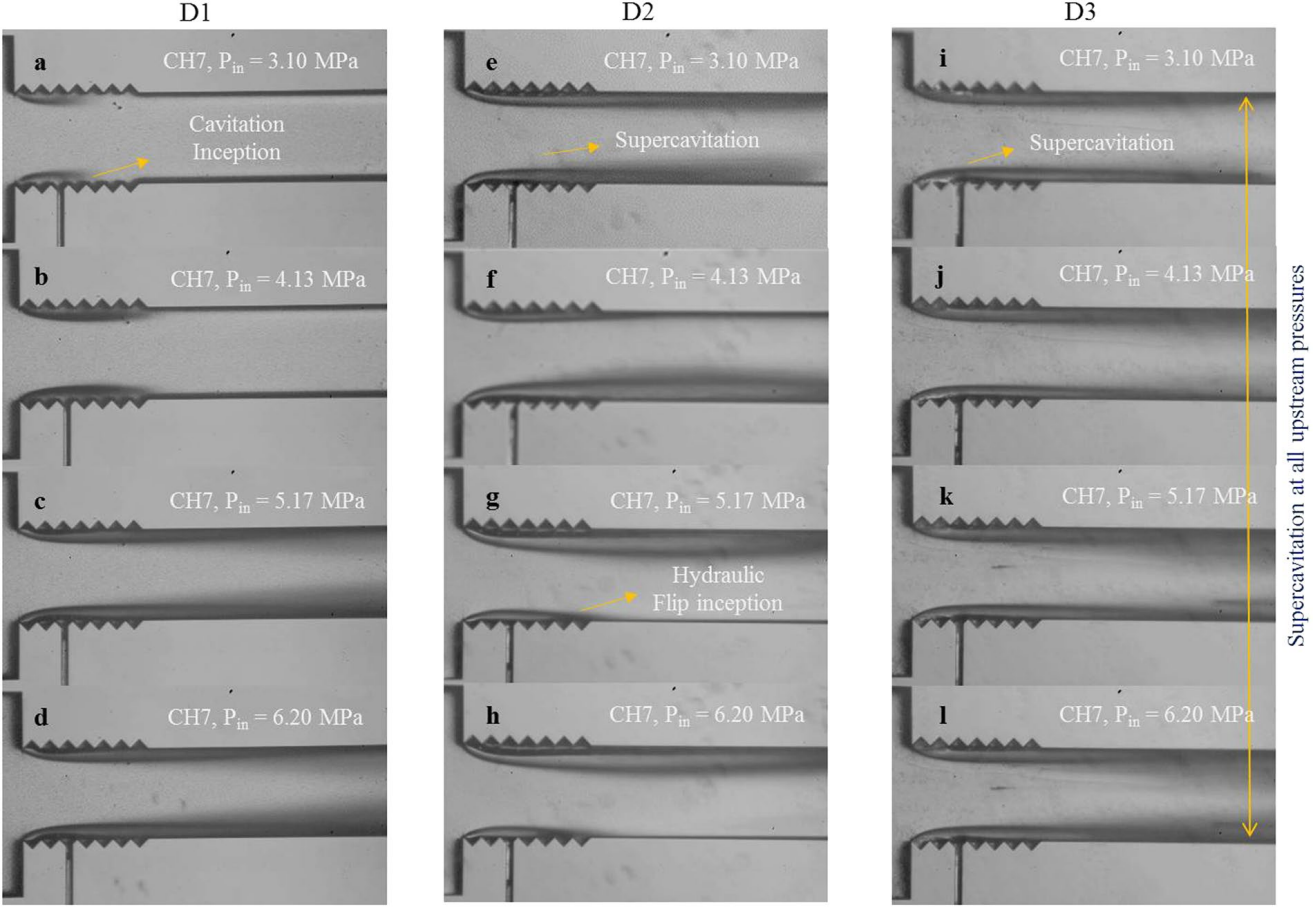
Comparison in the flow regimes inside CH7 at different upstream pressures for the D1, and D2 and D3 coatings.

Figure 9 illustrates the variations in cavitation number (σ) with respect to the vapor volume fraction (α_v_) for two big and small channels for the cases of D1 and D3. The dimensionless cavitation number is expressed as, ${\rm{\sigma }}={{\rm{P}}}_{{\rm{ref}}}-{{\rm{P}}}_{{\rm{v}}}/0.5{{\rm{\rho }}V}_{{\rm{ref}}}^{2}$, P_ref_ is reference pressure, which is the upstream pressure in this study, P_v_ is vapor saturation pressure and V_ref_ is the reference velocity at the outlet of the microchannel, which is calculated using the mass flow rate. Here, vapor volume fraction is found with the aid of the liquid, vapor and mixture densities. The latter is obtained by dividing mass flow rate by volumetric flow rate. The mass flow rate is obtained by measuring the mass of the fluid within the specified time period. The microchannels with SLIPS have lower cavitation numbers and higher values of vapor volume fractions (Fig. 9). The results show that the smaller microchannels (CH3 and CH5) for both cases of D1 and D3 have higher values for the vapor volume fraction, while the larger microchannels (CH1 and CH7) have instead higher cavitation numbers. This suggests that although the larger microchannels offer earlier inception and supercavitation, the smaller microchannels generate more intense cavitation and denser vapor phase specifically at higher upstream pressures (Fig. 10). As a result, in spite of more intense cavitating flows for the smaller microchannels, the larger microchannels require less input energy regarding cavitating flows.

## Concluding Remarks

In this study, SLIPS is presented as an effective method for earlier generation of cavitating flows, which is essential to decrease the input energy and increase the efficiency in microfluidic devices utilizing bubble clouds. While a less intense and earlier cavitation inception is observed inside the smaller microchannels (CH5 and CH6), more intense inception is captured in the extended channel region. The results show that the supercavitation condition is visible for all the microchannels for the D3 coating (with SLIPS), while this flow regime cannot be even recorded in some of the plain surface microchannels including the larger ones (e.g. CH2 and CH4). In addition, the supercavitation condition can be seen at a much lower upstream pressure for the D3 coating. Another interesting pattern, hydraulic-flip, which has not been seen in micro scale before, is observed at upstream pressures higher than 4.13 MPa for the D2 coating (layer-by-layer assembly of silica nanoparticles).

The findings of this study will assist in facile cavitating flow generation with the aid of the cheap and facile surface modification techniques as well as in the utilization of cavitating flows in biomedical and energy applications.

## Methods and Materials

### Methods and materials for creating SLIPS

#### Materials

Tetraethyl orthosilicate (TEOS), ammonia solution 25%, 2-propanol 99.5% (IPA), poly(sodium 4-styrenesulfonate), average molecular weight of 70,000 (SPS), poly(allylamine hydrochloride), average molecular weight of 50,000 (PAH), 1 H,1 H,2 H,2H-perfluorodecyltriethoxysilane 97% (PFDTS) were purchased from Sigma-Aldrich (St. Louis, USA). Fomblin Y LVAC 25/6, average molecular weight of 3,300 (PFPE) was purchased from Solvay (Brussels, Belgium). Deionized water (resistance 18.2 MΩ cm) was obtained using Milli-Q.

#### Assembly of thin film coatings for SLIPS

NPs: Monodispersed silica nanoparticles were synthesized in parallel lines with the literature^25^ by tuning the ratio of [TEOS]/[NH_3_]_aq_ to control the size of the particles.

Preparing LbL solution: LbL assembly was performed in two different ways in order to assess the properties of thin films on silicon wafer and evaluate the generation of cavitating flows within the microfluidic devises. For applying the thin film both on bare silicon wafer and microfluidic devices, two solutions of positively charged PAH and a solution of negatively charged SPS with concentrations of 10 mM and pH of 4.00, 7.50 and 4.00, respectively, were prepared. Silica NPs dispersed in water were prepared as a mixture (1:1) of 40 and 80 nm number average size with concentration of 0.03 g/L each. The pH of the negatively charged suspension was adjusted to 7.50.

LbL assembly of thin films on microfluidic devices and bare silicon wafer: The assembly of the thin film on bare silicon wafer was performed using the dip coating technique, and the substrates, which were sonicated in glass cleaning solution for 15 mins and rinsed in water for another 15 minutes, were submerged in the oppositely charged polyelectrolytes and NPs for 10 minutes and were rinsed for 2/1/1 minute in three separated distilled water tubes.

**Figure 11:**
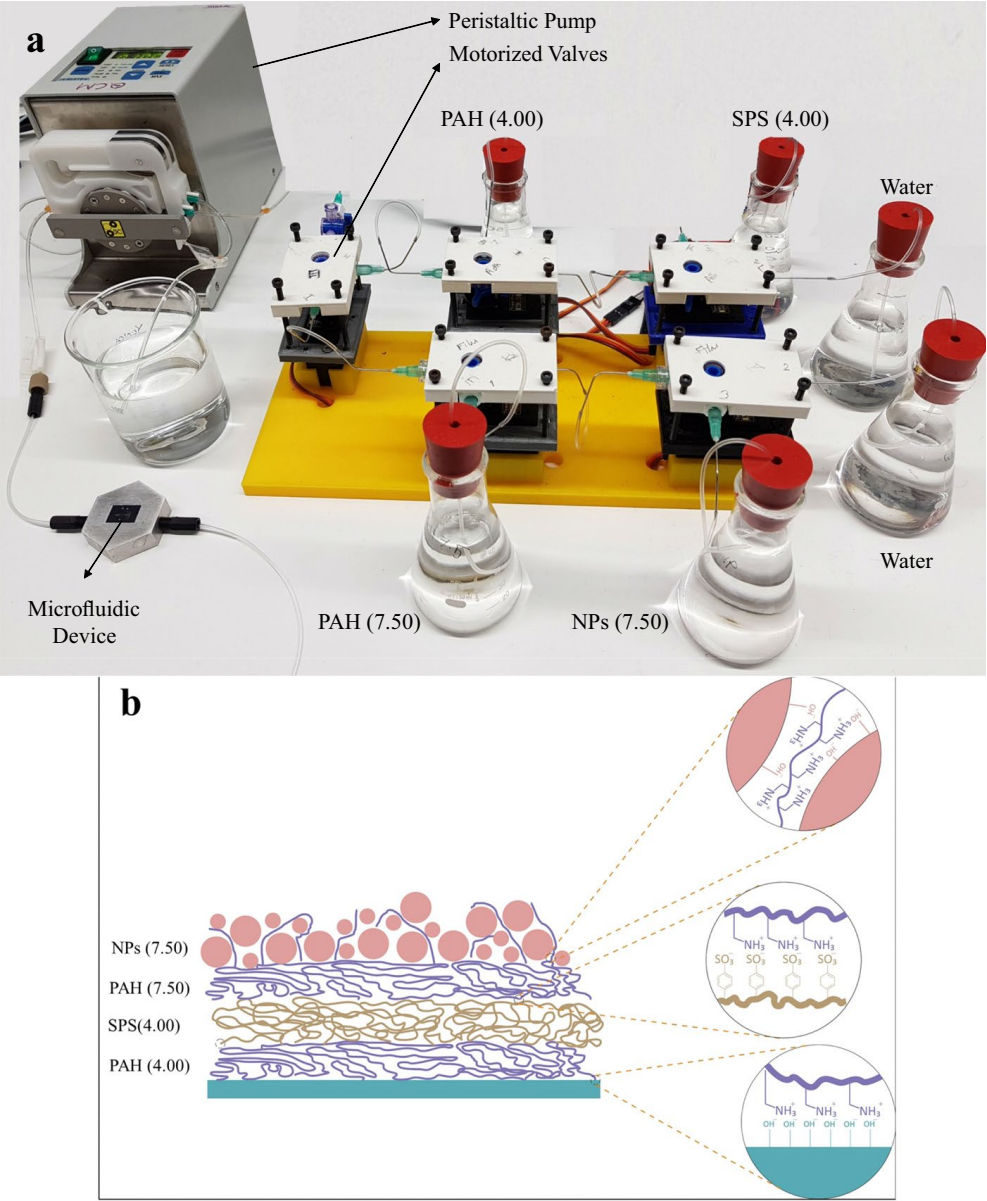
(**a**) Peristatic pump and a home-made Polyelectrolyte Distributer System (PDS) (**b**) schematic of Layer by Layer assembled polyelectrolytes and nanoparticles.

Microfluidic devices have an inaccessible geometry due to their design and high pressures, which they should withstand during cavitation tests. As it was impossible to apply the SLIPS before the fabrication of microfluidic devices, the assembly of thin film on devices was executed using a peristatic pump and a home-made polyelectrolyte distributer system (PDS) to pass the various polyelectrolytes consecutively with a feeding rate of 0.2 mL/s through the devices and then to rinse it with a sufficiently large quantity of water to remove excessive and loosely bounded electrolytes or NPs (Fig. 11a).

The final schematic of the thin film both on bare silicon wafers and microfluidic devices is as follows: (PAH(4.00)||SPS(4.00))5||(PAH(7.50)||NP(7.50))10 (Fig. 11b).

Heat treatment and applying lubricant: The prepared thin films for both cases were dried in room temperature and under a vacuum chamber (75 torr) for at least 12 hours. A solution of PFDTS and ethanol 1%(V/V) was applied on the surface of the thin films followed by another 12 hour period of 75 torr vacuum exposure in room temperature to evaporate and clear away the excessive ethanol from the surface. The samples were undergone a 2 hour heat treatment of 100 °C to increase both the durability and stiffness of the thin film and creation of covalent bonds between fluorine compound and thin film. Eventually, a very thick layer of Fomblin YL VAC 25/6 lubricant with an average molecular weight of 3300 a.m.u was spread over the thin film on the silicon wafer and was spun for 60 seconds at 1500 RPM to remove excess oil. For microfluidic devices, the lubricant was injected to the channel.

### Experimental procedure and configurations

All the microfluidic devices were fabricated out of double side polished silicon wafers with a thickness of 380 µm. All the steps of the fabrication of microfluidic devices were executed according to the techniques adopted from semiconductor based microfabrication techniques, which were explained in our previous study^3^. Seven microfluidic devices with different hydraulic diameters and side wall roughness length were employed in this study. The height of the side wall roughness elements was selected as 0.1D_h_. These elements were formed as a structural roughness according to the geometry design. The detailed properties of these devices were listed in Table 2. Meanwhile, the equivalent sand-grain roughness (k_s_) was calculated as 5.863 µm using the algorithm presented by Adams *et al*.^26^, which is suitable for low measured roughness values.

**Table 2:** The detailed properties of the microfluidic devices.

Microfluidic device	Microchannel hydraulic diameter, D_h_ [µm]	Side wall roughness Length
CH1	200	0.5 L
CH2	200	L
CH3	133	0.33 L
CH4	133	0.5 L
CH5	100	0.33 L
CH6	100	0.5 L
CH7	240	0.33 L

The flow restrictive element in the experimental setup presented in our previous study^3^ induces a sudden drop in pressure due to reduction in the cross-sectional area so that small bubbles from the flow restrictive element emerge due to hydrodynamic cavitation. It is predicted to obtain intense generation of cavitation bubbles with the surface modification. For all the microchannel configurations, the walls of microchannels are equipped with three pressure sensors to measure local pressures at the prescribed locations, namely before the entrance of the restrictive element, vena contracta (1D_h_) and entrance of the extended channel (5D_h_–8D_h_)^27^. The same sensors were used to assess the pressure recovery inside the microchannel.

The glass wafer assists in visualizing fluid flows inside the channels. The experiments were conducted by applying different inlet pressures. The inlet pressure was varied from 1 to 7.23 MPa, while the outlet pressure at the microchannel was fixed to 0.1 MPa. The whole steps of the visualization experiments were comprehensively explained in our previews study^28^. The visualization was carried out in two main regions. The first region was the entrance of the microchannel with the length of 1.6 mm, where the pressure was expected to decrease to the vapor saturation pressure, and the second region was the extension region with the width of 900 µm and length of 1.6 mm, where the bubble generation was likely to appear in this area.

### Characterization

#### DLS

The size and zeta potential of synthesized NPs were measured in a disposable capillary cell (DTS1070) using the Dynamic Light Scattering (DLS) technique via Malvern Zetasizer Nano ZS.

#### AFM and SEM

The surface topography was obtained using the Bruker Multimode 8 atomic force microscope (AFM). The height images were captured using NanoAndMore tips with a bending spring constant of 40 N m, resonance frequency of 50–200 kHz, and tip radius of 10–20 nm. All the images were processed (using procedures for plane-fit and flatten). The surface morphology of the samples was analyzed by field emission scanning electron microscopy (FESEM, LEO Supra VP-55).

#### CA

Contact angle (CA) and contact angle hysteresis (CAH) were measured using the equipment Attension Theta Lite. A 5 µL droplet of distilled water (18.2 MΩ·cm) was accumulated on the surface of the samples for measuring the CA, and by adding and removing the water from the stabilized droplet, the contact angle hysteresis was obtained. The reported values are the average of three subsequent measurements for each sample.

#### Ellipsometry

Thickness, reflectance, refractive index and porosity of thin films were measured and modeled using J.A. Woollam Co. M2000 ellipsometry. The measurements were conducted in the range of 380 to 780 nm (∼1.6–3.3 eV) wavelengths.
